# Walkability and walking for transport: characterizing the built environment using space syntax

**DOI:** 10.1186/s12966-016-0448-9

**Published:** 2016-11-24

**Authors:** Mohammad Javad Koohsari, Neville Owen, Ester Cerin, Billie Giles-Corti, Takemi Sugiyama

**Affiliations:** 1 Behavioural Epidemiology Laboratory, Baker IDI Heart and Diabetes Institute, Melbourne, Australia; 2 Faculty of Sport Sciences, Waseda University, Saitama, Japan; 3 Institute for Health & Ageing, Australian Catholic University, Melbourne, Australia; 4 McCaughey VicHealth Centre for Community Wellbeing, Melbourne School of Population and Global Health, University of Melbourne, Melbourne, Australia; 5 School of Health Sciences, Swinburne University of Technology, Melbourne, Australia

**Keywords:** Space syntax, Walkability, Urban design, Walking, Street layout, Built environment, Urban form

## Abstract

**Background:**

Neighborhood walkability has been shown to be associated with walking behavior. However, the availability of geographical data necessary to construct it remains a limitation. Building on the concept of space syntax, we propose an alternative walkability index, space syntax walkability (SSW). This study examined associations of the full walkability index and SSW with walking for transport (WT).

**Methods:**

Data were collected in 2003–2004 from 2544 adults living in 154 Census Collection Districts (CCD) in Adelaide, Australia. Participants reported past week WT frequency. Full walkability (consisting of net residential density, intersection density, land use mix, and net retail area ratio) and SSW (consisting of gross population density and a space syntax measure of street integration) were calculated for each CCD using geographic information systems and space syntax software. Generalized linear models with negative binomial variance and logarithmic link functions were employed to examine the associations of each walkability index with WT frequency, adjusting for socio-demographic variables.

**Results:**

Two walkability indices were closely correlated (ρ = 0.76, *p* < 0.01). The associations of full walkability and SSW with WT frequency were positive, with regression coefficients of 1.12 (95% CI: 1.08, 1.17) and 1.14 (95% CI: 1.10, 1.19), respectively.

**Conclusions:**

SSW employs readily-available geographic data, yet is comparable to full walkability in its association with WT. The concept and methods of space syntax provide a novel approach to further understanding how urban design influences walking behaviors.

## Background

Physical activity and sedentary behaviors are recognized as determinants of chronic disease risk [1, 2]. Given the limited success of individually-based approaches to behavior change (e.g., motivation, guidance, and education), attributes of the built environments where people live and work are now understood to be important potential determinants of active living [3]. In conceptualizing aspects of urban form that may be relevant to physical activity and subsequent health outcomes, a landmark study by Cervero and Kockelman [4] proposed the concept of 3Ds: Density, Diversity, and Design, and examined how these constructs are related to travel behavior. They found that neighborhoods with high population density, diverse land uses, and pedestrian-oriented design were more likely to facilitate active travel choices, which can contribute significantly to the overall physical activity [4].

The concept of the 3Ds was further extended to develop the construct of ‘neighborhood walkability’, which consists of residential density (Density), land use mix (Diversity), intersection density (Design), and net retail area ratio (Design) [5]. Often, walkability is calculated as the sum of these four components, e.g., as the sum of standardized scores [5] or as the sum of decile scores [6]. Walkability has been found to be associated with walking behavior not only in the U.S.A. [7], but also in Australia [8], Canada [9], and Belgium [10]. However, the difficulty of collecting the relevant geographical data remains a limitation in calculating walkability. Net residential density, land use mix, and net retail area ratio require parcel-level information about land use and retail floor area (for net retail area ratio), which is often unavailable or difficult to source [11–13]. Reliance on detailed spatial data can be an impediment to the application of walkability to practice and decision making in urban design.

A concept of “space syntax” has considerable potential in developing a walkability index that is less data-intensive and easier to produce. Space syntax is fundamentally concerned with street network, but it is also known to be related to functional aspects of urban form, including land use [14]. Below, we describe the concept of space syntax and how one of the space syntax measures, integration, could substitute other walkability components in constructing a new walkability index.

**Fig. 1:**
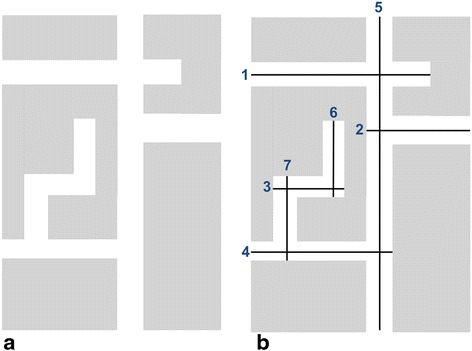
**a** A schematic diagram of a hypothetical neighborhood and **b** its axial lines (numbers represent segment names)

**Fig. 2:**
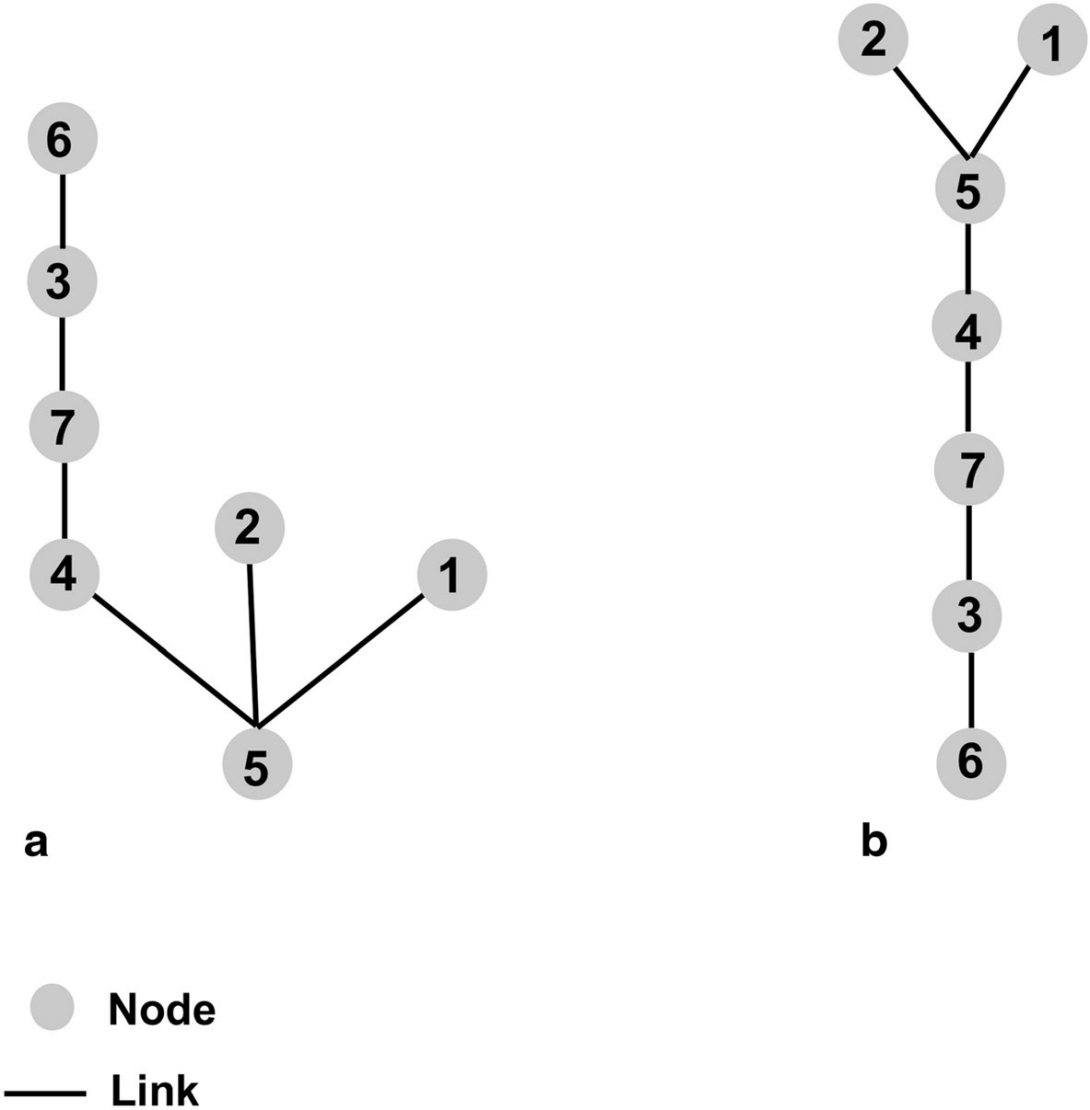
Justified graphs using node 5 (**a**) and node 6 (**b**) as the base node

**Fig. 3:**
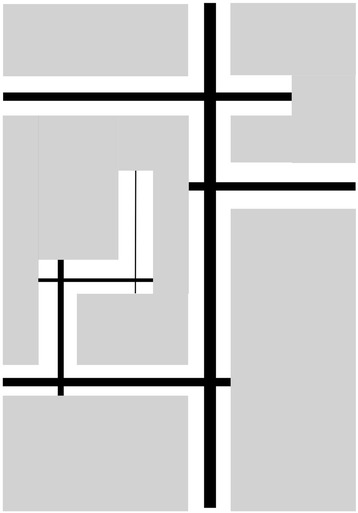
Level of integration (*thicker lines* represent higher levels of integration)

Space syntax is a concept and method that has been developed primarily in the fields of urban design and architecture, in order to understand impact of the spatial configuration of urban areas and buildings on people’s movement [15]. The fundamental building block of space syntax is “axial lines” that represent lines of sight [16]. At the urban scale, axial lines correspond to street segments [16], and space syntax is concerned with the topological (relational) aspect of the street network, i.e., how axial lines are connected to each other. Figure 1 shows (a) a neighborhood schematic and (b) its axial lines. All space syntax measures are calculated using a “justified graph”, which shows diagrammatically how each axial line (“node”) is connected to its adjacent axial lines (whereby a connection is regarded as a “link”) [17]. Figure 2 shows the justified graphs for the neighborhood represented in Fig. 1, with nodes 5 and 6 as the base nodes. “Depth” is calculated from the justified graph as “the sum of the links that must be traversed if one were to move from that space [street] to all other spaces [streets]” [18]. Mean depth is the sum of the number of links from each node to the base node divided by the total number of nodes minus one [15]. For node 5 (Fig. 2a), the mean depth is 2.0 (= (1 + 1 + 1 + 2 + 3 + 4)/6). For node 6 (Fig. 2b), the mean depth is 3.3 (= (1 + 2 + 3 + 4 + 5 + 5)/6). Integration is a key measure for space syntax, which is the inverse of the mean depth: the lower the mean depth (less street segments to be traversed to reach the segment in question), the higher the integration. It indicates how topologically “close” a street segment is to the other segments within the network [19]. In this context, integration is not about a metric distance between segments, but is the sum of turns required in moving from one segment to another. Compared with less-integrated segments, more-integrated street segments require fewer turns to reach the segment from other streets, thus is considered to be more accessible [19–21]. In contrast, intersection density is a simple count of intersections within a unit area. Thus, it is possible that two neighborhoods having the same intersection density can have different levels of integration. Figure 3 shows the levels of integration for streets in the neighborhood presented in Fig. 1.

Space syntax measures are related to pedestrian movement. Several empirical studies have shown positive correlations between integration and the presence of pedestrians [22–24]. A potential factor explaining the link between higher street integration and more pedestrians is land use along street segments. Commercial land uses may exist along highly-integrated streets, because such streets are more accessible from other locations, which is important for commercial land uses [25]. It can be thus argued that more integrated street segments attract more pedestrians partly because of the presence of commercial destinations along them [25]. A recent empirical study supports this argument: it shows a significant association between integration and walking for transport (WT), with 42% of the total effect of integration on WT being accounted for by a measure of commercial destination availability [14]. Hillier and colleagues have argued that street layout is the “primary generator of pedestrian movement” [25]. This means that street network, which is essentially a formal aspect of urban form, could influence pedestrian movement through differential distribution of commercial land uses according to the level of integration.

**Fig. 4:**
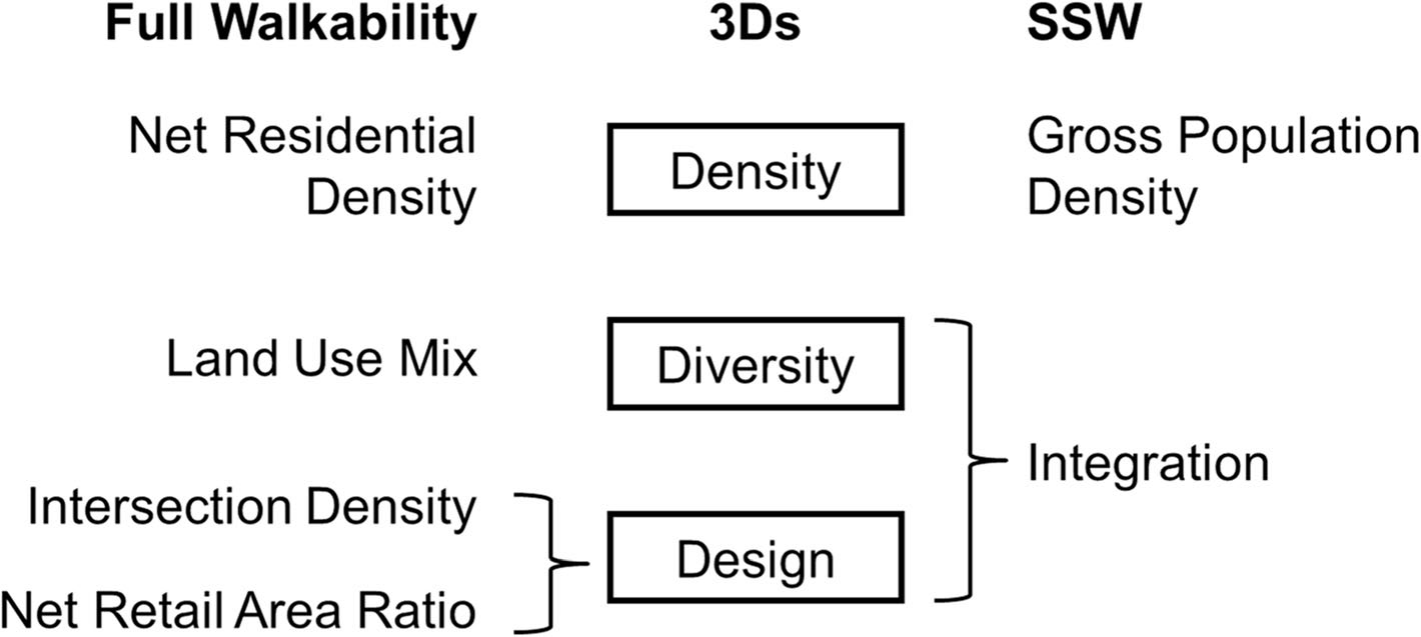
Conceptual diagram showing how the concept of 3Ds are operationalized in full walkability and SSW

Building on the theory of space syntax and empirical studies using integration, we propose “space syntax walkability” (SSW). Figure 4 shows how the construct of the 3Ds is operationalized in full walkability and in SSW.

Full walkability is a composite of net residential density (Density), land use mix (Diversity), intersection density (Design), and net retail area ratio (Design), whereas SSW consists of gross population density (Density) and integration (Diversity and Design). We used gross population density, which does not require land use data, thus is easier to calculate compared to net residential density.

Our aim is to examine concurrently the associations of full walkability and SSW with WT, in order to evaluate the utility of the proposed SSW index.

## Methods

### Data source

Data were from the Physical Activity in Localities and Community Environments (PLACE) study conducted in Adelaide, Australia (population: 1.1 million) during 2003–2004. Detailed methods of study design and sampling procedures have been described elsewhere [8]. Briefly, residential addresses were randomly selected from 154 census collector districts (CCD, a geographical unit comprising of about 250 households) stratified based on full walkability and area-level socioeconomic status (SES) within the Adelaide Statistical Division. These CCDs had a median size of 23 hectares (range: 5.2–251.8 hectares). An invitation letter to participate in the study was sent to the addresses identified within each CCD, and a self-administered survey was sent to those who were eligible (20–65 years) and agreed to participate. The total number of respondents was 2650 (11.5% of the residential addresses initially identified).

#### Measures

##### Walking for transport (WT)

Frequency of WT was used as the outcome in this study. Participants reported their frequency of WT (days) in the past week, using the following question: “During the last 7 days, on how many days did you walk for at least 10 min at a time to go from place to place?”. This is an item in the International Physical Activity Questionnaire-Long Form (IPAQ) [26]. Adequate reliability and validity of this instrument have been previously reported [26]. Frequency of walking instead of duration was used because of known over-reporting of duration in this instrument [27]. Recent studies have also used walking frequency, due to similar concerns about the accuracy of the walking duration measure [28, 29].

##### Full walkability

Full walkability was calculated for each CCD as a composite measure consisting of objectively-determined net residential density, intersection density, land use mix, and net retail area ratio. Net residential density was the ratio of the number of dwelling units to the land area for residential use within each CCD. Cadastral (parcel) data from the 2001 South Australian Digital Cadastral Data and the 2001 South Australia Land Ownership and Tenure System were used to calculate residential area [6]. Intersection density was calculated as the ratio of the number of intersections (3-way or more) to the land area of a CCD (square kilometers) using street centreline data from the South Australian Department of Transport. Land use mix was defined as an entropy index describing the heterogeneity of five land uses (residential, commercial, recreational, industrial, and other) within a CCD [30]. Net retail area ratio was the ratio of the retail floor space to the retail parcel area. Land use mix and net retail area ratio measures were calculated using land use, zoning data, shopping center location data and census data for the Adelaide Statistical Division, obtained from the South Australian Government Department for Transport and Urban Planning, and the 2001 Adelaide Retail Database [6]. Data required to calculate them (parcel-level land use and retail building footprint) are difficult to obtain, even in high income countries such as the U.S.A. [31, 32]. Full walkability was calculated using the following formula developed by Frank et al. [5]:

$$ \mathrm{Full}\ \mathrm{walkability} = \mathrm{z}\ \left[\mathrm{z}\ \left(\mathrm{n}\mathrm{e}\mathrm{t}\ \mathrm{residential}\ \mathrm{density}\right) + 2\times \mathrm{z}\ \left(\mathrm{intersection}\ \mathrm{density}\right) + \mathrm{z}\ \left(\mathrm{retail}\ \mathrm{floor}\ \mathrm{area}\ \mathrm{ratio}\right) + \mathrm{z}\ \left(\mathrm{land}\ \mathrm{use}\ \mathrm{mix}\right)\right], $$

where “z (*variable*)” indicates a standardized (z) score of the *variable*.

##### Space syntax walkability (SSW)

SSW was calculated as a composite measure of gross population density and integration. Gross population density was the ratio of the number of residents to the land area of each CCD, which were derived from the 2001 Australian census data. Integration was calculated using street centerline data and Axwoman [33] and DepthMap (University College London, London) software. Axwoman is a free extension of ArcGIS that auto-generates axial lines from street centerline data [16]. Then, the axial lines were imported into the DepthMap software, in which integration for each street segment is calculated following the procedure discussed above [34]. An integration score was assigned to each street segment considering all the other streets within 1 km of the center of the segment. For each CCD, the mean integration score was calculated for all street segments within the CCD. SSW was calculated using the following formula:

$$ \mathrm{S}\mathrm{S}\mathrm{W} = \mathrm{z}\ \left[\mathrm{z}\ \left(\mathrm{gross}\ \mathrm{population}\ \mathrm{density}\right) + 2\times \mathrm{z}\ \left(\mathrm{integration}\right)\right]. $$

Please note that integration was weighed twice to be consistent with the formula used for full walkability.

##### Socio-demographic attributes

Participants were asked to report their age, gender, educational attainment, work status, marital status, having children in the household, and annual household income. SES of each CCD was also identified using its median household weekly income, and all CCDs were dichotomized into a lower or higher SES category using the median.

#### Statistical analyses

Spearman’s correlation coefficients between four walkability components were calculated. A series of regression analyses were conducted to estimate the strength of associations of full walkability and SSW with WT. We first compared how each density measure was associated with WT (Model 1: net residential density, Model 2: gross population density), then examined associations of each street network measure with WT (Model 3: intersection density, Model 4: integration). Full walkability and SSW were examined separately in Model 5 and 6. Since the outcome variable (WT frequency) consisted of positively-skewed count data, generalized linear models with negative binomial variance and logarithmic link functions were employed to examine the associations of each exposure measure with WT frequency, adjusting for socio-demographic variables (income, age, sex, employment status, household composition, marital status, and CCD-level SES).

All exposure measures were standardized to allow comparison between models. Robust standard errors were used to account for clustering of participants within each CCD. Associations were expressed in the form of antilogarithms of regression coefficients and their 95% confidence intervals (CI), indicating the proportional difference in the frequency of WT associated with a 1 SD increment in a specific walkability component or index. Akaike Information Criterion (AIC) and Bayesian Information Criterion (BIC) were used as measures of model fit. Smaller AIC and BIC values are indicative of better fitting models. Stata 11.0 (Stata Corp, College Station, Texas) was used to conduct the analyses.

## Results

After excluding those who did not provide information on WT frequency, data from 2591 participants were analyzed. Participants reported a mean of 3.2 days of WT in the past week (SD = 2.5). Table 1 shows the characteristics of the study sample. Table 2 shows the correlation matrix between all environmental measures for 154 CCDs. Correlation coefficients between walkability components (items 1 to 6) were mostly significant and positive, ranging from 0.3 to 0.7, except for non-significant and negative associations involving land use mix. No significant correlations were observed between land use mix with intersection density and integration. The correlation between the full walkability and SSW was 0.76 (*p* < 0.01). SSW was correlated positively with all walkability components except for land use mix.

**Table 1:** Characteristics of study participants (*N* = 2591)

Variable	Mean (SD) or N (%)
Age (years)	44.4 (12.3)
*Missing*	14 (0.5%)
Gender	
*Women*	1652 (63.8%)
*Missing*	5 (0.2%)
Employed	
*Yes*	1649 (63.6%)
*Missing*	0 (0.0%)
Education	
*Tertiary or higher*	1192 (46.0%)
*Missing*	0 (0.0%)
Children in household	
*Yes*	794 (30.6%)
*Missing*	0 (0.0%)
Marital status	
*Single*	1086 (41.9%)
*Couple*	1432 (55.3%)
*Other*	73 (2.8%)
*Missing*	0 (0.0%)
Household income (AUD$ per annum)	
*< $20800*	595 (23.0%)
*$20800–41599*	650 (25.1%)
*$41600–77999*	729 (28.1%)
*≥ $78000*	503 (19.4%)
*Missing*	114 (4.4%)
Days of WT in the past week	3.2 (2.5)

**Table 2:** Spearman’s correlation coefficients between walkability components and walkability indices

	1	2	3	4	5	6	7	8
1. Net residential density	1	0.60**	−0.41**	0.30**	0.73**	0.42**	0.56**	0.57**
2. Intersection density		1	0.01	0.49**	0.61**	0.60**	0.87**	0.68**
3. Land use mix			1	0.26**	−0.28**	0.12	0.27**	0.00
4. Net retail area ratio				1	0.36**	0.67**	0.78**	0.64**
5. Gross population density					1	0.52**	0.57**	0.77**
6. Integration						1	0.73**	0.93**
7. Full walkability							1	0.76**
8. SSW								1

**p < 0.01

Table 3 shows the results of the negative binomial regression models, examining associations of net residential density, gross population density, intersection density, integration, full walkability and SSW with the frequency of WT. The associations of all density and street network measures with WT frequency were positive, with (antilogarithms of) regression coefficients ranging from 1.09 to 1.12. For full walkability and SSW, an increment of 1 SD unit in these indices was associated with 12% (95% CI: 8%, 17%) and 14% (95% CI: 10%, 19%) higher frequency in WT, respectively. The best fitting model (smallest AIC and BIC values) was observed for SSW. However, the differences in model fit were very small.

**Table 3:** Associations of density, street layout and walkability measures with WT frequency

Model	Exposure	IRR (95% CI)	AIC	BIC
1	Net residential density	1.09 (1.05, 1.13)**	4.614239	−17967.44
2	Gross population density	1.12 (1.08, 1.16)**	4.610708	−17976.57
3	Intersection density	1.09 (1.05, 1.14)**	4.613614	−17969.06
4	Integration	1.12 (1.08,1.16)**	4.609942	−17978.55
5	Full walkability	1.12 (1.08, 1.17)**	4.609867	−17978.75
6	SSW	1.14 (1.10, 1.19)**	4.607357	−17985.24

All models accounted for clustering at the CCD level and adjusted for age, gender, education, marital status, children in household, income, employment status, and neighborhood SES. All exposure measures were standardized

*IRR* incidence rate ratio

*AIC* Akaike information criterion

*BIC* Bayesian information criterion

**p < 0.01

## Discussion

This study found that full walkability (a composite of four components) and SSW (a composite of two components) were closely correlated, despite them being constructed in conceptually different ways, and both indices were positively and equally associated with the frequency of WT. These results suggest that SSW could be a surrogate of full walkability, which would have practical utility when parcel-level land use and retail area data are not available. There has been an attempt to address the issue of data availability in producing a walkability index. An Australian study examined how an abridged walkability (3 components without net retail area ratio) was associated with walking in comparison to the full four-component walkability [35]. It found that both the abridged and the full walkability indices were similarly associated with the prevalence of walking to work. The present study examined a closely-related issue, but employed an innovative approach, using a measure of space syntax to characterize diversity and design, two of the three underlying constructs of walkability.

Although both the full walkability and SSW indices were similarly related to walking, SSW offers several advantages. First, compared with full walkability, SSW can be calculated using more readily-available geographical data. Gross population density is easier to obtain than is net residential density, which requires data on land use. Street centerline data, which are commonly available through local government authorities, are the only data required to calculate integration. Actual calculation of integration can be conducted in DepthMap, which is a free software program developed by University College London [34]. In contrast, full walkability requires parcel-level land use data and retail building floor area data. This leads to difficulties in constructing the full walkability index and is a significant impediment to comparing findings across different contexts, which could differ in the way land uses are defined. SSW appears to be a simpler yet effective alternative to full walkability, with the potential to be used in other urban areas where parcel-level data are not available, and by a broad range of organizations and practitioners, including local governments, urban designers, and developers.

Correlation coefficients shown in Table 2 indicate that integration was not associated with land use mix. It was initially anticipated that integration was associated with land use mix, because areas with highly-integrated streets are expected to have more commercial destinations than those with less-integrated streets [14]. However, high land use mix may not necessarily indicate the presence of commercial destinations. For instance, areas that are equally divided into residential, industrial, and recreational land uses can be high in land use mix, without having retail destinations. The negative correlation between net residential density and land use mix may suggest that commercial land uses, which can be expected to exist near high residential density areas, may not necessarily play a key role in determining the land use mix variable. On the other hand, high correlation between integration and net retail area ratio was observed. High net retail area ratio means tightly-built retail areas (less space for car parking within a retail parcel), which are easy to access by foot. Our findings suggest that integration can capture the presence of ‘walkable’ retail destinations. The correlation coefficient between integration and street connectivity was not high (ρ = 0.60). This is because these two measures capture different aspects of the street network.

Our study has several limitations. The self-report measure of WT may be subject to recall error, although we focused on frequency – walking instances that may be easier for study participants to recall – rather than on the duration of walking. Walking may have happened outside the areas within which walkability was determined. Location-specific measures of walking (e.g., walking to/from home) are needed to accurately assess the relationships between walkability measures and local walking. The self-selection issue may also play a role: those who prefer to walk may have chosen to live in high-walkable neighborhoods. All road segments (which would be inclusive of highways) were used to calculate the integration measure. Although producing pedestrian street networks is time consuming and expensive, they provide a more accurate picture on how pedestrians can traverse the network. Using a new mapping technology (e.g., Google maps), further studies can focus on pedestrian networks to calculate street network measures [36]. Walkability and its components were calculated for each CCD, which differed widely in size. Further research using walkability indices, calculated for each participant using a buffer area is needed to confirm the findings of the study. In addition, the way in which street centerlines are represented in GIS (e.g., the use of double or single line for streets with a median strip) may influence the street network measures. The low response rate may also have introduced selection bias. The study was conducted in the urban areas of Adelaide. As such, the findings of this study may have been partly due to the particular spatial characteristics of this city, thus may not be applicable to other localities. In order to confirm the usability of SSW, further studies in different geographical contexts are warranted.

Compared with intersection density, the space syntax measure of integration is less intuitive and thus may be more difficult to grasp for practitioners and decision makers. Identifying how best to explain and communicate this for these important constituencies should be a priority. However, it is notable that the walkability indices so far developed are also measures based on abstract components such as land use mix. The advantage of space syntax (e.g., the ease of getting the necessary geographic data, its utility of capturing multiple aspects relevant to pedestrians) may outweigh this limitation. Another advantage of space syntax is that it can identify connectivity not only for an area but also for a single street segment. Future research using space syntax should examine the level of integration that may be sufficient for an area or a street to support walking trips.

## Conclusions

Our study suggests that the concept of space syntax and associated methods can be employed to produce an easy-to-calculate indicator of walkability. An alternative walkability index developed in this study, SSW, may be used, for example, in developing countries or other settings where land use data are not easily available. Space syntax measures are now used to investigate relationships between urban form and issues relevant to pedestrians such as crime and wayfinding [37, 38]. Further applications of space syntax can help advance research on the built environment, physical activity and health.

## Abbreviations

CCD: Census collection districts
SES: Socioeconomic status
SSW: Space syntax walkability
WT: Walking for transport
